# Gene Lifestyle Interactions With Relation to Obesity, Cardiometabolic, and Cardiovascular Traits Among South Asians

**DOI:** 10.3389/fendo.2019.00221

**Published:** 2019-04-09

**Authors:** Shafqat Ahmad, Syeda Sadia Fatima, Gull Rukh, Caren E. Smith

**Affiliations:** ^1^Department of Medical Sciences, Molecular Epidemiology, Uppsala University, Uppsala, Sweden; ^2^Preventive Medicine Division, Harvard Medical School, Brigham and Women's Hospital, Boston, MA, United States; ^3^Department of Nutrition, Harvard T. H. Chan School of Public Health, Boston, MA, United States; ^4^Department of Biological and Biomedical Sciences, Aga Khan University, Karachi, Pakistan; ^5^Department of Neuroscience, Functional Pharmacology, Uppsala University, Uppsala, Sweden; ^6^Nutrition and Genomics Laboratory, Jean Mayer U. S. Department of Agriculture Human Nutrition Research Center on Aging, Tufts University, Boston, MA, United States

**Keywords:** gene environment interaction, South Asians, obesity, cardiometabolic traits, cardiovasclar disease

## Abstract

The rapid rise of obesity, type 2 diabetes mellitus (T2DM) and cardiovascular disease (CVD) during the last few decades among South Asians has been largely attributed to a major shift in lifestyles including physical inactivity, unhealthy dietary patterns, and an overall pattern of sedentary lifestyle. Genetic predisposition to these cardiometabolic risk factors may have interacted with these obesogenic environments in determining the higher cardiometabolic disease prevalence. Based on the premise that gene-environment interactions cause obesity and cardiometabolic diseases, we systematically searched the literature and considered the knowledge gaps that future studies might fulfill. We identified only seven published studies that focused specifically on gene-environment interactions for cardiometabolic traits in South Asians, most of which were limited by relatively small sample and lack of replication. Some studies reported that the differences in metabolic response to higher physical activity and low caloric diet might be modified by genetic risk related to these cardiometabolic traits. Although studies on gene lifestyle interactions in cardiometabolic traits report significant interactions, future studies must focus on more precise assessment of lifestyle factors, investigation of a larger set of genetic variants and the application of powerful statistical methods to facilitate translatable approaches. Future studies should also be integrated with findings both using mechanistic studies through laboratory settings and randomized clinical trials for clinical outcomes.

## Overview

About 20% of the world's population lives in the South Asian region comprised of Bangladesh, Bhutan, India, Nepal, Pakistan, and Sri Lanka (1). The burden of obesity (2), type 2 diabetes mellitus (T2DM) (3), cardiovascular disease (CVD) (4) is especially high in South Asian populations, partly because South Asians appear to be more genetically susceptible to CVD than, for example, white Europeans(5, 6). For example, the prevalence of CVD ranges from 5 to 10% in South Asians vs. 1.2 in % Caucasians (6); where age, male gender, smoking history, abnormal lipids, hypertension, and diabetes are considered as the major risk factors in both groups (7). CVD is projected to become the leading cause of mortality and morbidity among South Asians in the coming 20 years (8). Similarly, 8–15% of individuals in India (9), 26.0% in Bangladesh (10), 13–14% in Sri Lanka (11), and 19% in Nepal (12) suffer from obesity; another alarming trigger for cardiometabolic and CVD pathogenesis. South Asians have several distinct features with respect to T2DM risk compared with other ethnic groups, including an earlier onset of disease and complications (13), occurrence at a lower BMI (2), insulin resistance (14), ectopic fat deposition (15), and younger age (16); therefore the World Health Organization (WHO) recently proposed an ethnicity based BMI cut-off to predict T2DM and CVD (17). South Asian countries are also shifting rapidly from traditional agricultural lifestyles to industrialized living, the health care infrastructure of many South Asian countries is under-developed and South Asian populations are aging rapidly (18, 19). Thus, there is a pressing need to understand the etiology of cardiometabolic and CVD at a global level, but in developing countries the urgency is even greater.

South Asians have experienced a considerable shift to a more automated lifestyle with fewer physically active behaviors and more energy dense foods, but there is a considerable variability in the extent to which obeso-genic environments increase the risk of cardiometabolic and CVD related disease. These differences in response have been reported in randomized clinical trials (20) as well as migration studies (21, 22), reflecting the impact of environment on cardiometabolic traits and CVD, while also supporting an important role of genetics. These literature findings strongly suggest that the development of cardiometabolic and CVD related disease is the consequence of interactions between genetic and environmental risk factors.

The Human Genome Project (HGP) was designed to map human genome with the identification of more than 20,500 human genes (23, 24). The products of this project provide details about the structure, function, physical location and organization of human genome, including differences in minor allele frequency and linkage equilibrium related to ancestry. After the successful sequencing of 3 billion nucleotides through HGP, the main focus was placed on the identification and cataloging of millions of SNPs. The knowledge from HGP made GWAS possible and contrasts with the conventional candidate gene approach in that it is performed in a fashion that does not rely on previous knowledge about genetic variants and traits. The GWAS approach is considered most unbiased approach for discovering genetic variants that affect susceptibility to disease.

GWAS studies focus on investigating the association between common genetic variants and traits within populations. Earlier GWAS studies led to the discovery of *FTO* rs9939609 genetic variant in association with obesity among Europeans (25–27). After this early wave of GWAS studies, a major shift in GWAS studies took place that resulted in collaboration between geneticists to facilitate pooling of study specific data through meta-analysis. The increase in statistical power that accompanied these large studies ultimately led to the detection of small association signals. Regarding obesity traits, the formation of the ‘Genetic Investigation of ANthropometric Traits (GIANT), a large international consortium, is the most prominent example. In one of the first efforts of the GIANT consortium, GWAS summary statistics of 16,876 participants from seven European ancestry studies were meta-analyzed and led not only to the confirmation of *FTO* rs9939609 variant but also identified *MC4R* rs17782313 genetic variant in association with BMI (28). In the recent wave of discovery by the efforts of GIANT consortium, GWAS has led to the identification of 97 BMI-associated and 49 BMI adjusted waist-hip ratio associated reproducible genetic variants (29, 30). In an effort to discover low frequency (<5% minor allele frequency) variants, Turcot et al. used a sample of 718,734 individuals to identify 14 protein coding variants (31). Pulit et al. using a sample of 694,649 individuals identified 463 genetic variants that associate with BMI adjusted waist-hip ratio (32).

Similar collaborative efforts involving pooling of GWAS summary statistics data regarding T2DM and CVD-related traits led to the foundation of the MAGIC (Diabetes Genetics Replication and Meta-analysis) and the GLGC (Global Lipids Genetics Consortium), consortia, respectively. In the recent wave of discovery by the efforts of the MAGIC consortium, more than 65 genetic variants associated with glycemic traits have been identified (33, 34). Kooner et al. identified six T2DM associated genetic loci in a sample of 18,731 cases of T2DM and 39,856 disease free individuals from South Asia (35). Similarly, GWAS collaborative efforts of GLGC consortium led to publication of 157 robustly associated genetic variants in association with CVD related traits (36, 37). These genetic variants discovered though GWAS explain <5% of genetic variation in obesity (29, 30), <15% in lipid traits (36, 37), and <11% in T2DM related traits (34). Estimates for the heritability of obesity, T2DM and CVD mostly range from 40 to 70%, showing that a large proportion of genetic risk still remained to be discovered. While the specific genetic mutations that lead to the high heritability estimates observed for obesity, cardiometabolic and CVD have been studied extensively in Europeans (29, 30, 33, 34, 36–39), very few GWAS studies have been reported in indigenous South Asian populations. An even smaller number of studies have used gene environment approaches to investigate the high prevalence of CVD and its risk factors in South Asians.

The study of gene environment interactions provides useful insight to investigators across several disciplines. Recognition that genes interact with the environment to influence anthropometric, cardiometabolic and CVD traits is highly relevant to public health research. For geneticists, it is also important to understand how lifestyle factors interact with genetic predisposition to affect gene expression, function, regulation, and other genetic features that lead to the development of a disease. In addition, studying gene-environment interactions is important as it helps to optimize health interventions by targeting the individuals who respond well, can reduce unnecessary intervention side effects, and also can identify novel interventions that are specifically effective in a population subgroup of genetically similar people.

The GWAS approach has been very successful in identifying the common genetic variants. Follow-up of genetic variants that were discovered through GWAS to investigate gene environment interactions is generally very feasible, as these genetic loci are usually genotyped in huge epidemiological samples which also contain extensive lifestyle and clinical information. However, even with large epidemiological samples, only a few genetic variants that were discovered through conventional GWAS approach have shown convincing evidence that lifestyle factors can modify the anthropometric (40) and cardiometabolic disease risk (41) in Europeans. Just to reiterate, the objective of the current literature review was not to compare different statistical methodological approaches for discovering potential genetic variants for studying gene environment interactions which has been discussed elsewhere (42).

## Objective of This Review

In this review we intend to discuss the published studies in South Asians regarding gene and lifestyle factors including physical activity, smoking, and dietary patterns interactions in the development of obesity, cardiometabolic and CVD disease related biomarkers of insulin resistance, glucose metabolism, lipids, blood pressure, and inflammation.

### Methodology

A literature search was performed on studies related to obesity, cardiometabolic and CVD related diseases with respect to interaction of genes and lifestyles including physical activity, smoking, sleep disorders, and dietary intakes in PubMed and Google Scholar. We carried out a literature search using the term gene environment in South Asians, as has been described in the Supplementary Note. For the current study we only identified seven original published articles (not the published reviews) (as shown Table 1) that investigated gene environment interactions in obesity, T2DM and CVD related traits among South Asians. We also searched the published articles by the name of genetic epidemiologists working explicitly with cardiometabolic and cardiovascular related traits among South Asians.

**Table 1:** List of studies published regarding gene-environment interactions in obesity, cardiometabolic, and cardiovascular diseases among South Asian.

**Authors**	**Study type**	**Sample (*N*=)**	**Study population**	**Interactor variable**	**Genetic variants**	**Outcome**
Ahmad et al. (43)	Case-control	16,157	South Asian	Physical activity	97 BMI associated SNPs	BMI
Ahmad et al. (44)	Case-control	14,131	South Asian	smoking	GWAS, FLJ35541 genetic variant	BMI
Reddon et al. (45)	Cross-sectional	2760	Multi-ethnic including South Asians	Physical activity	FTO	Obesity
Merrit et al. (46)	Cross-sectional	174	Multi-ethnic including South Asians	Dietary protein intake	FTO	Body weight
Vimaleswaran et al. (47)	Cross-sectional	1618	South Asians	lifestyle factors	FTO	Metabolic traits
Ayyappa et al. (48)	Case control	1845	South Asian	High fat diet	LPL (lipoprotein lipase)	HDL-C
Bodhini et al. (49)	Cross-sectional	1682	South Asian	Dietary fat intake and PA	TCF7L2 and MC4R	HDL-C

#### Gene Environment Interactions for Obesity Among South Asians

The high prevalence of obesity is believed to be the result of genetic susceptibility and lifestyle factors and more than 200 interaction studies have been reported in Europeans (40). Among South Asians, Ahmad et al. (43) performed one of the first studies using data from 16,157 Pakistani adults of Pakistan Risk of Myocardial infarction (PROMIS) study, and studied the interactions between 95 BMI associated genetic variants with physical activity for the risk of obesity. The authors reported that out of 95 BMI-associated genetic variants discovered in Europeans, 73 were directionally associated with the risk of BMI in South Asians. However, these 95 BMI-associated variants explained less (1.54%) of the phenotypic variance in BMI among South Asians compared to 2.7% among Europeans. In the overall Pakistani sample each unit increase in the genetic risk score for BMI was associated with 0.04 kg/m^2^ increase in BMI per allele; *P* = 4.5 × 10^−14^). The authors also observed nominal significant interactions of *GALNT10* rs7715256 (*P*_interaction_ =0.048), *CADM2* rs13078960 (*P*_interaction_ = 0.037), and *CLIP1* rs11057405 (*P*_interaction_ = 0.014) with physical activity, while *HIP1* rs1167827 (*P*_interaction_ = 0.015), *C6orf106* rs205262 (*P*_interaction_ = 0.032), *PTBP2* rs11165643 (*P*_interaction_ = 0.045), and *GRID1* rs7899106 (*P*_interaction_ = 0.043) genetic variants showed significant interactions with smoking on the risk of obesity. Although interactions with individual variants were observed, the authors did not observe any evidence of significant interactions between the BMI-specific genetic risk score with physical activity or smoking on the risk of obesity.

Recently, the Toronto Nutrigenomics and Health Study investigated the interactions between dietary macronutrients and variants in the *FTO* gene using a cross-sectional sample of 1,639 different ancestries sample including 174 South Asians individuals. The authors reported no association between *FTO* rs1558902 variant with the risk of adiposity in South Asians (46). The authors also observed that protein intake did not modify the genetic risk of the same *FTO* variant on adiposity, as had been reported in Europeans. The study sample is relatively small and might be underpowered to detect any reliable genetic association or gene diet interactions in South Asians. Moreover, this example illustrates the critical need for larger samples of South Asians for genetic studies.

Similarly, Reddon et al. (45) using the longitudinal, multiethnic cohort of EpiDREAM comprised of 17,423 subjects including 2,760 individuals of South Asian ancestry reported that vigorous physical activity ameliorated obesity in genetically susceptible subgroups. The authors reported significant interactions between physical activity and *FTO* rs1421085 genetic variant on the risk of BMI (*P*_interaction_ = 0.03). One of the caveats of the study was that it did not report ethnic-specific gene diet interactions despite having a multiethnic sample.

Several of the studies described above use the GWAS approach which has been very successful in identifying associations between common genetic variants and disease traits but less able to identify gene lifestyle interactions. The heterogeneity of variance (HeVa) approach extends the conventional GWAS approach by taking into account the trait variance across three different genotypes of a biallelic single nucleotide polymorphism (SNP), which reflects a different mode of genetic influence regarding interactions. HeVa approach tests whether interactions are present by testing a trait significantly differs across three genotypes of a SNP locus (50, 51). So, in that way HeVa is preferred over the GWAS approach in detecting the potential candidates for gene environment interactions, although it needs to be remembered that heterogeneity can be driven through gene-gene or other interacting factors, such as lifestyle. By using the HeVa approach in the sample of 14,131 individuals of PROMIS study from Pakistan, Ahmad et al. identified *FLJ33534* rs140133294 variant in association with the variance of BMI and also the same variant interacted with smoking (*P*_interaction_ = 0.0005) (44). The HEVA approach has been considered as one of the powerful tools for discovering potential gene environment interactions. However, recently Shungin et al. reported that efficiency of potential targets for gene environment interactions could be improved by focusing genetic variants with low *p*-values discovered through marginal GWAS effects in combination with the HEVA approach (52).

#### Gene Environment Interactions in Type 2 Diabetes Among South Asians

In addition to the growing number of studies investigating BMI in South Asians, other groups have focused on T2DM. Vimaleswaran et al. (47) using a sample of 734 T2DM prevalent cases and 884 disease free participants from the Chennai Urban Rural Epidemiology Study (CURES) reported a novel gene diet interaction between genetic variants and diet for obesity traits and diabetes in an Asian Indian population. The authors selected two *FTO* associated genetic variants (rs8050136 and rs11076023). The findings suggested that minor allele carriers of *FTO* rs8050136 who consumed high carbohydrate had higher risk of developing obesity traits (47). The authors also observed a significant interaction between *FTO* rs11076023 genetic variant and dietary fiber intake (*P*_interaction_ = 0.0008) where individuals carrying AA genotypes with higher fiber intake had 1.62 cm lower waist circumference than among those carrying the TT genotype. Further, the authors observed that physically inactive individuals carrying the A allele of rs8050136 *FTO* SNP had 1.89 times significantly increased risk of obesity compared to those with CC genotype (*P*_interaction_ = 4.0 × 10^−5^).

#### Gene-Environment Interactions in CVD Related Traits Among South Asians

Ayyappa et al. (48) analyzed the association of four (rs285, rs328, rs4922115, and rs1121923) lipoprotein lipase gene (*LPL*) associated variants with blood lipids. The authors investigated whether dietary fat intake can modify the genetic risk of abnormal lipids in a sample of 1,845 individuals comprised of 788 T2DM prevalent cases and 1,057 healthy participants from India. The authors observed significant interactions between *LPL* rs1121923 genetic variant and dietary fat intake on HDL (*P*_interaction_ = 0.003), specifically that among consumers of a high fat diet, individuals carrying T allele of rs1121923 had higher HDL-C levels.

Bodhini et al. (49) used a cross-sectional study of 861 prevalent cases of T2DM and 821 normally glucose tolerant participants from the Chennai Urban Rural Epidemiology Study (CURES). The authors examined whether the association of *MC4R* rs17782313 and *TCF7L2* (rs12255372 and rs7903146) gene variants and cardiometabolic traits could be modified by physical activity and dietary intake. Authors observed significant interactions between *TCF7L2* rs12255372 and dietary fat intake on the outcome of HDL-C (*P*_interaction_ = 0.0001). Low total dietary fat intake positively modifies the HDL-C levels in this Asian Indian population, despite their carrying the risk genotype (GT + TT) of *TCF7L2* rs12255372. In sensitivity analyses authors also observed significant interactions between poly unsaturated fatty acids and the *TCF7L2* rs12255372 variant on HDL-C (*P*_interaction_ < 0.0001). This finding is of public health significance given that among Asian Indians low fat intake improves HDL-C among those carrying higher genetic risk for low HDL-C, which puts them at markedly increased risk for CVD (49).

## Challenges in Gene-Environment Interactions

Gene lifestyle interaction studies can be very effective in disentangling the biological pathways for predicting disease development. These studies can also address the problem of missing heritability in several ways. First, interaction studies may identify cardiometabolic-related genetic variants which exert their effects more visibly through interaction with different lifestyle exposures. Second, gene lifestyle interactions can also be used for identifying the environmental factors that affect persons with specific genotypes (53). However, it remains a major challenge to identify interactions in cardiometabolic and cardiovascular diseases due to considerable limitations including imprecise measurement of lifestyle factors, small sample size, and limited statistical power, meta-analytic combinations of many small sample studies with heterogeneous lifestyle assessment and lack of replication [as has been described elsewhere (40, 54)]. Ideally, in order to reduce the probability of false positive findings, a sample comprising thousands of participants is required for gene environment interaction studies which unfortunately is not available for many studies. Recent efforts in the establishment of large population based prospective cohorts (e.g., UK Biobank Study [https://www.ukbiobank.ac.uk/], Million Veteran Program [https://www.research.va.gov/mvp/], Estonian Genome Centre [https://www.geenivaramu.ee/en/about-us/estonian-genome-centre] and Finnish Precision Medicine Initiatives have provided robust and repeated measurements of lifestyle exposures over time in European populations. The UK biobank sample, in particular, has already led to some of the robust examples of gene lifestyle interactions in cardiometabolic traits (55–58). However, in South Asian populations, it still remains a challenge to conduct gene lifestyle interaction studies as there are only a few studies with relatively large samples. However, there are many existing epidemiological cohorts which can potentially be genotyped for studying gene-lifestyle interactions in cardiometabolic traits among South Asians. Determination of cardiometabolic disease-specific epigenetic, transcriptomic, proteomics, and human microbiota changes and integration of that information with genetic and lifestyle factors will provide more comprehensive insights and will prove valuable in predicting the onset and progression of obesity, cardiometabolic, and cardiovascular diseases.

## Conclusions

Evidence shows that complex traits including obesity, cardiometabolic and CVD result from interaction between multiple genetic and environmental factors. During the last decade, the GWAS has led to identification of a number of genetic variants that associate with obesity, cardiometabolic and CVD traits, however, these genetic variants only explain a small phenotypic variation in these traits. Identifying genetic variants that predispose to cardiometabolic traits in combination with specific lifestyle exposures might be important for understanding the disease etiology and subsequently providing better prevention and treatment strategies. The identification of gene lifestyle interactions may also help in disentangling the pathways involved in these complex diseases and will be beneficial for future drug development and therapy. Currently, the study of gene lifestyle interactions in cardiometabolic traits among South Asians has been challenged by insufficient knowledge, small sample size, and lack of replication compared to other ancestries i.e., Caucasians. The next generation of studies incorporating large epidemiological cohort studies utilizing genetic information and better environment measurement technologies can improve our understanding of the complex causes of obesity, cardiometabolic, and CVD diseases.

It is also important to recognize that, similar to the international collaborative efforts in the European cohorts, South Asian studies have taken the initiative to form collaborations to fill the knowledge gaps in terms of interaction studies between genetic and lifestyle factors. As these efforts continue to progress, more personalized strategies can be implemented in decreasing the burden of these increasingly prevalent non-communicable diseases.

## Author Contributions

SA: contributed to the conception and design of the research; SA, SF, GR, and CS: contributed to the acquisition, analysis, and interpretation of the data; SA: drafted the manuscript; All coauthors critically revised the manuscript, agree to be fully accountable for ensuring the integrity and accuracy of the work, and read and approved the final manuscript.

### Conflict of Interest Statement

The authors declare that the research was conducted in the absence of any commercial or financial relationships that could be construed as a potential conflict of interest.
